# 

**Published:** 1891-06

**Authors:** 


TO CONTRIBUTORS.

Articles for publication must reach this office not later than 15th inst.; and are expected to be published exclusively in this JOURNAL. Extra copies of JOURNAL furnished contributors when requested. Reprints by special arrangement. Publication of an article does not necessarily imply endorsement of the views therein expressed.

MEDICAL AND SURGICAL JOURNAL, Post-office box 431.

